# A new species of *Megischus* Brullé (Hymenoptera, Stephanidae) from China, with a key to the Chinese species

**DOI:** 10.3897/zookeys.69.738

**Published:** 2010-10-18

**Authors:** Chun-dan Hong, Cornelis van Achterberg, Zai-fu Xu

**Affiliations:** 1 Department of Entomology, College of Natural Resources and Environment, South China Agricultural University, Guangzhou 510640, P. R. China; 2 Department of Terrestrial Zoology, Netherlands Centre for Biodiversity Naturalis, Postbus 9517, 2300 RA Leiden, Netherlands

**Keywords:** Hymenoptera, Stephanidae, *Megischus*, new species, China

## Abstract

A new species of Megischus Brullé from China, Megischus aplicatus **sp. n.**,is described and illustrated. A key to the Chinese species of Megischus is added. The holotype is deposited in the Parasitic Hymenoptera Collection of Zhejiang University, Hangzhou.

## Introduction

The genus Megischus Brullé, 1846 (Hymenoptera: Stephanidae) is cosmopolitan, but most species are known from the Indo-Australian region (van Achterberg 2002). In total, 82 species of the genus Megischus were previously known worldwide (Aguiar 2004, 2006;  van Achterberg and Yang 2004; van Achterberg and Quicke 2006). For the Chinese fauna, only two species were recognized: Megischus ptosimae Chao, 1964 and Megischus chaoi van Achterberg, 2004. Megischus ptosimae was reared from Ptosima chinensis Marseul, 1867 (Coleoptera: Buprestidae) on peach trees (Chao 1964) and from Buprestidae on other Prunus species (van Achterberg and Yang 2004).

The genus Megischus is characterized mainly as follows: body medium sized to large; temple without conspicuous ivory stripe along outer orbit; forewing with four or more closed cells; first subdiscal cell of fore wing comparatively slender and vein 2-1A completely pigmented; vein 1-M of fore wing 2.2–8.0 times as long as vein 1-SR; hind femur with two large ventral teeth; hind tarsus of female 3-segmented and of male 5-segmented; ovipositor sheath with an ivory subapical band (van Achterberg 2002).

## Material and methods

The specimen was collected in Hubei Province, China, and is deposited in the Parasitic Hymenoptera Collection of Zhejiang University, Hangzhou (ZJUH).

Morphological terminology, including the wing venation system, follows van Achterberg (2002). Descriptions were made under an Olympus SZ61 stereoscope, in combination with a 40W LED lamp. Photographic images were processed with both Image-Pro Plus and AnalySIS Extended Focal Imaging software, and figures were finished with ACDSee10.0 and Photoshop CS 8.0.1, mostly to adjust the size and background.

## Results

### Genus Megischus

Brullé, 1846

Megischus Brullé 1846: 537. Type species: Megischus annulatorBrullé 1846 (designated by Viereck 1914) [= Stephanus furcatus (Lepeletier & Serville, 1825)].
Megischus Brullé 1846: van Achterberg 2002: 53–168; Aguiar and Johnson 2003: 469–482.
Bothriocerus Sichel 1860: 759. Type species: Bothriocerus europaeus Sichel, 1860 (by monotypy) (= Stephanus anomalipes Foerster, 1855, according to Madl 1991).

#### Key to species of the genus Megischus Brullé from China.

**Table:** 

1	Gena narrowly rounded medially behind eye in dorsal view (Fig. 18 in van Achterberg and Yang 2004); neck postero-dorsally at about same level as middle part of pronotum (Fig. 23, l.c.); vein cu-a of fore wing strongly reclivous (Fig. 24, l.c.); hind basitarsus about 4 times as long as wide; [without distinct pronotal fold and without a cavity; vein 1-M of fore wing about 5 times as long as vein 1-SR and 1.2 times vein m-cu; widened part of hind tibia of female nearly straight or weakly concave ventrally (Fig. 20, l.c.); ivory part of ovipositor sheath about twice as long as dark apical part]	Megischus chaoi van Achterberg, 2004
–	Temple medially roundly convex behind eye in dorsal view (Fig. 25 in Van Achterberg and Yang 2004); neck at lower level than middle part of pronotum postero-dorsally (Fig. 29, l.c.); vein cu-a of fore wing weakly reclivous or subvertical (Fig. 26, l.c.; 7); hind basitarsus 3.0–3.5 times as long as wide	2
2	Temple slightly convex behind eye (Fig. 25 in Van Achterberg and Yang 2004); pronotal fold distinct and with a cavity below it (Figs 27–29, l.c.); vein 1-M of fore wing 4.2–5.5 times as long as vein 1-SR and 1.1–1.3 times vein m-cu; first tergite largely transversely striate or striate-rugose; head largely blackish or dark brown; widened part of hind tibia of female distinctly concave ventrally (Figs 30, 31, l.c.), but straight in male; [whitish or ivory part of ovipositor sheath 0.7–2.0 times as long as dark apical part]	Megischus ptosimae Chao, 1964
–	Temple distinctly convex behind eye (Fig. 3); pronotal fold absent (Fig. 4–5); vein 1-M of fore wing about 2.2 times as long as vein 1-SR and 0.9 times vein m-cu (Fig. 8); first tergite largely smooth and shiny dorsally (Fig. 9); head largely orange brown; widened part of hind tibia of male nearly straight ventrally (Fig. 10)	Megischus aplicatus sp. n.

#### Megischus aplicatus

Hong, van Achterberg & Xu sp. n.

urn:lsid:zoobank.org:act:65A9A7F9-83C9-4D9E-ADC5-D058A6C44656

Figs 1 12 

##### Male.

Length of body 25.7 mm, and of fore wing 13.9 mm.

###### Head.

**Figures 1–7.**
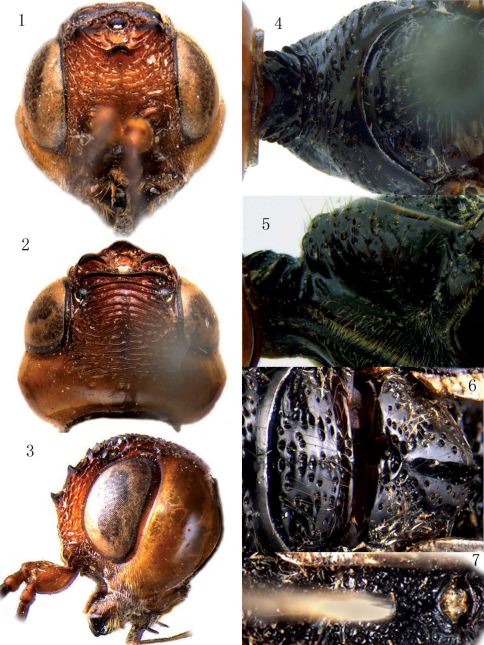
Megischus aplicatus sp. n. ♂. **1** head, anterior view **2** head, dorsal view **3** head, lateral view **4** pronotum, dorsal view **5** pronotum, lateral view **6** mesoscutum and scutellum **7** propodeum.

Antenna with 41 segments; first antennal segment 1.6 times as long as wide and twice as long as second segment, third segment 2.4 times as long as wide and 0.8 times as fourth segment; frons (Fig. 1) strongly rugose, rugae laterally curved upwards; three anterior coronal teeth large, two posterior ones connected and somewhat sinuate; vertex (Fig. 2) with 4 strongly curved carinae, followed by irregularly transversely striate area, striae coarser laterally and largely interrupted medio-dorsally, resulting in a more or less longitudinal impression, sculpture disappearing near occipital carina, leaving a narrow smooth area; area along inner orbit with one distinct longitudinal carina on each side; temple (Fig. 3) largely smooth and shiny, roundly convex occipital carina strongly developed.

###### Mesosoma.

Neck (Fig. 4) rather short and robust, medio-dorsally rather shallowly concave, laterally with pairs of strong carinae, neck at much lower level than middle part of pronotum; pronotal fold absent (Fig. 4–5); middle and posterior part of pronotum strongly punctate dorsally and laterally, punctures bearing setae and with smooth interspaces; lateral oblique groove of pronotum smooth and shallowly impressed (Fig. 5), ventral area below it punctate and setose; propleuron coriaceous and densely setose; prosternum irregularly punctate, punctures posteriorly more dense and with setae; mesoscutum (Figs 6) shiny, foveolate and with smooth interspaces, laterally foveolate, largely coalescent, areolate; notauli and median groove indistinct and formed by some small foveolae; axillae foveolate and setose; scutellum (Fig. 6) medially largely smooth and laterally sparsely foveolate; mesopleuron robust, dorsal part finely setose, convex part evenly punctate and with smooth interspaces, each puncture bearing a whitish seta, metapleuron medially distinctly convex and densely foveolate-rugose, with fine setosity, ventral part largely smooth and with both dorsal anterior depression and ventral one rather deep; propodeum (Fig. 7) densely reticulate-foveolate.

###### Wings.

**Figures 8–12.**
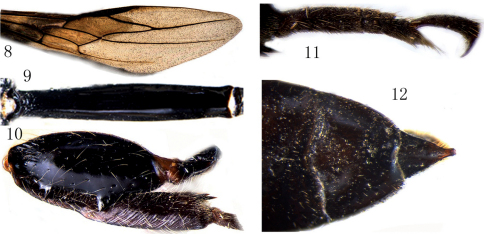
Megischus aplicatus sp. n. ♂. **8** fore wing **9** first tergite **10** hind femur and tibia **11** hind tarsus **12** pygidial process.

Fore wing (Fig. 8): vein 1-M distinctly curved, 2.2 times as long as vein 1-SR and 0.9 times vein m-cu; vein 2-SR 1.1 times as long as vein r; vein r ends 0.5 times length of pterostigma behind level of apex of pterostigma; vein 1-SR 0.95 times as long as parastigmal vein; vein cu-a postfurcal and subvertical; vein 3-CU1 largely nebulous.

###### Legs.

Hind coxa rather strong, annular, coarsely punctate and setose; hind femur (Fig. 10) sparsely punctate and with whitish setae, area in between smooth and shiny, ventrally with two large teeth and some denticles in between; hind tibia robust, ventrally mostly straight and 1.3 times as long as hind femur, basal narrow part about 0.6 times as long as widened part, outer side obliquely carinate, inner side apically densely setose; hind basitarsus (Fig. 11) subparallel-sided, 3.5 times as long as its apical width and 4.7 times as long as second tarsus.

###### Metasoma.

First tergite largely smooth and shiny (Fig. 9), 5.2 times as long as its maximum width, 1.4 times as long as second tergite and 0.5 times as long as remainder of tergites; remainder of tergites smooth; pygidial process (Fig. 12) distinct and tubular apically.

##### Colour.

Head orange brown; pronotum, mesosoma, first tergite and hind legs largely dark brown or black; metasoma except first tergite brown to blackish; wing membrane light brownish, wing venation and pterostigma dark brown.

##### Material examined:

Holotype male, China: Hubei, Shennongjia National Nature Reserve, viii.1982, Coll. Shi Shang-bo, No. 870112 (**ZJUH**).

##### Female.

Unknown.

##### Host.

Unknown.

##### Distribution:

China (Hubei).

##### Comments:

The new species runs in the key by van Achterberg (2002) combined with the revision by van Achterberg and Yang (2004) to Megischus ptosimae Chao. It differs as indicated in the included key to Chinese species.

##### Etymology:

The name of this species derives from the Latin “a-” and “plicatus” which means without fold, because this species has no pronotal fold on the pronotum.

## Supplementary Material

XML Treatment for Megischus
